# A community-based participatory protocol to improving communication with Black men about oral and pharyngeal cancers: Research protocol

**DOI:** 10.1371/journal.pone.0288478

**Published:** 2023-08-17

**Authors:** Patrick D. Smith, Marcus Murray, Tiosha Bailey, Caryn E. Peterson, Osei Bekoe, Darien J. Weatherspoon

**Affiliations:** 1 Division of Prevention and Public Health Sciences, University of Illinois Chicago College of Dentistry, Chicago, Illinois, United States of America; 2 Project Brotherhood, Chicago, Illinois, United States of America; 3 Tiosha Bailey Consulting, Chicago, Illinois, United States of America; 4 Department of Epidemiology and Biostatistics, University of Illinois Chicago School of Public Health, Chicago, Illinois, United States of America; 5 University of Illinois Cancer Center, Chicago, Illinois, United States of America; 6 Department of Dental Public Health, University of Maryland School of Dentistry, Baltimore, Maryland, United States of America; Universal Scientific Education and Research Network, CAMEROON

## Abstract

Black men are disproportionately impacted by oral and pharyngeal cancer (OPC) mortality. This is in part due to a lack of information received about OPCs and their associated risk factors during health encounters. Discussions between dentists and Black men may improve Black men’s knowledge, screening, and treatment uptake. Yet, dentists do not commonly communicate with Black men about OPCs due to their own discomfort. This paper describes the protocol for our research project, which proposes an initiative, grounded in community-based participatory research, to adapt a culturally-specific OPC communication tool. This tool will be adapted using a mixed-methods approach to assess the knowledge, attitudes, and experiences of Black men discussing OPCs and associated risk factors with dental providers. The tool will then be assessed for feasibility and acceptability among Black men, as well as dental students and dental providers in community-based clinical settings.

## Introduction

In the United States (U.S.), Black men have the highest mortality from oral and pharyngeal cancers (OPCs) compared to men with other racial/ethnic identities, including non-Hispanic whites, non-Hispanic Asians, and Hispanics, despite not having the highest incidence [1–3]. The elevated risk of Black men’s mortality from OPCs are mainly due to Black men being diagnosed at later stages of cancer progression and not receiving the same types of aggressive treatment recommendations as other population cohorts, regardless of the stage of diagnosis [4–6]. Minimal knowledge about OPCs and OPC risk factors, limited access to dental care, fear of dentists, pride, fatalism, and mistrust of dental providers have been cited as contributing factors to Black men’s lack of awareness about OPC risk, screening, and treatment [7–9]. Another reason why Black men may lack awareness about OPCs is that dentists may not routinely engage in OPC discussions or screening with them. Guidelines for oral cancer screening in the U.S. are unclear [10–13], and dentists may not participate in OPC discussions and/or screening due to their own discomfort, limited clinical time and insurance reimbursement, a lack of confidence in their own knowledge, training, and experience, and their anticipation of negative reactions from patients [14, 15].

Since the early 2000s, there has been national attention to address OPC prevention and mortality among Black men in the U.S [16]. Yet, to the best of our knowledge, there are no published reports of targeted communication interventions. The purpose of this paper is to describe the research protocol for a community-based participatory research initiative aimed to improve the quality of dental provider participation in OPC discussions and screening among Black men. Healthy People, an initiative sponsored by the U.S. Office of Disease Prevention and Health Promotion to monitor the nation’s progress in several key health indicators, has set a target to increase the proportion of oral and pharyngeal cancers detected at the earliest stage from 29.5% to 34.2% by 2030 [17]. Thus, establishing communication strategies for dental providers to address oral cancer among Black men has promise for improving Black men’s knowledge about OPCs and screening uptake [18].

**Fig 1:**
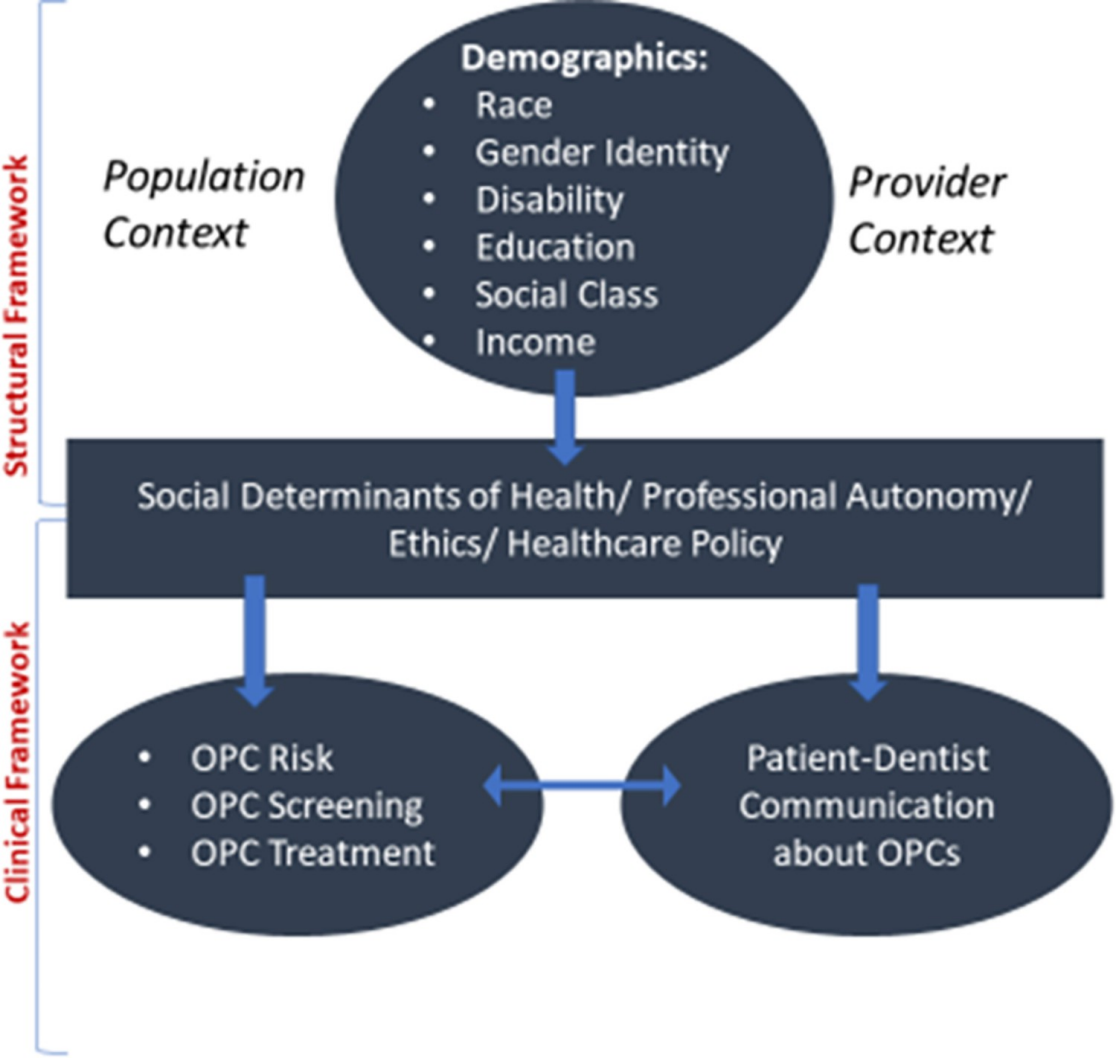
Illustration of the conceptual framework for Black men and dental provider misalignment.

This research will engage community stakeholders to identify the socioecological context for Black men’s health and share those contexts with dental providers through the communication tool, to facilitate improved OPC prevention, risk reduction, and dental care workforce capacity. The hypothesis for this research is that the OPC communication tool will help to: (1) improve dental providers’ knowledge about the social and structural contexts surrounding Black men’s OPC knowledge and experiences seeking dental care; (2) improve the self-efficacy and confidence of dental providers to communicate with Black men about OPC risk, screening, and treatment; and (3) improve Black men’s knowledge and uptake of OPC screening. What is innovative about this study is that it will leverage community partnerships and expertise to incorporate the attitudes, beliefs, and life experiences of Black men to systematically determine how OPC preventive care is delivered to them. This concept is described by the conceptual model (described below), which describes how clinical interactions between Black men and dental providers may or may not occur due to upstream, structural racism (Fig 1) [19–22].

## Methods

This protocol has been approved by the Institutional Review Board at the University of Illinois Chicago (Protocol # 2022–0658). To conduct this research, we will use community engagement principles partnering with Project Brotherhood, an organization based in Chicago, IL with a history of addressing Black men’s mistrust of healthcare providers and research institutions, to facilitate Black men’s participation in cancer screening and treatment [23]. The project consists of three aims that will be assessed in a stepwise approach. We describe the primary components of our study methodology using the Consolidated criteria for reporting qualitative research (COREQ) [24].

### Study design

For each aim, we will use a mixed-methods approach with a cross-sectional study design. All qualitative study components, including focus group sessions, will be led by an investigator experienced in qualtitative research methodology. Aim one consisted of focus groups and surveys to assess knowledge, attitudes, beliefs, and behaviors related to OPC screening among Black men within the Project Brotherhood network in metropolitan Chicago, IL. After focus group and survey data were analyzed, an advisory panel of focus group participants, researchers, and healthcare professionals with expertise in OPCs, dental practice, and/or patient communication was convened to adapt a pre-existing OPC communication tool that is specific to the needs and concerns of the study participants, identified through focus group and survey results [25]. This panel meets at least once per month to discuss focus group findings and develop ideas for implementing clinical guidelines for dentists’ communication with Black men about OPCs. Up to twenty of the original focus group participants will be invited back to receive a presentation of the tool and provide an assessment of the tool’s content, feasibility, and potential to improve communication among Black men and dental providers. This assessment will consist of a guided group discussion. For aim two, Project Brotherhood will lead an evaluation of the usability and effectiveness in improving provider self-efficacy of the OPC communication tool by facilitating a simulation training of predoctoral dental students enrolled at University of Illinois (UIC) Chicago College of Dentistry. Aim 3 will consist of a pilot test of the OPC communication tool in several community-based dental clinics in Cook County, IL to assess Black men’s perceptions, knowledge and experiences with dental providers who will be trained to use the tool in clinical settings.

### Settings

The project will take place in Chicago, IL. The focus groups conducted in aim one took place virtually. Aim two study activities will take place in-person, via a classroom and clinic simulation at UIC College of Dentistry. Aim three study activities will take place at select community-based dental clinic sites that are to be determined in the future.

### Periods

Initial focus groups for aim one of the project occurred from August 2022 to December 2022. The dental student evaluation of the OPC communication tool will take place from January 2024 to December 2024. Aim three, which is the community site evaluation of the OPC communication tool, will occur from January 2025 to December 2025.

### Inclusion and exclusion criteria

Inclusion criteria for the aim one focus groups were up to twenty-five individuals who identified as Black men, regardless of cis or trans identity, who are within the Project Brotherhood network in metropolitan Chicago, IL. Women were excluded from the study because the oral cancer disparity of interest is specific to Black men. It should also be noted that the focus groups targeted individuals who self-identified as men, regardless of whether they identified as cis or trans. Aim two will recruit up to fifty participants who must be predoctoral students enrolled in the dental education program at UIC College of Dentistry. There are no exclusions for pre-doctoral dental students who meet the inclusion criteria. For participation in aim three, dental providers must be licensed to practice dentistry in Chicago, IL and be employed by a community-based clinic with a contractual affiliation with UIC. There are no exclusions for dentists who meet the inclusion criteria. Additionally, men who participate as patients in the piloting of the communication tool must only identify as Black men, regardless of cis or trans identity.

### Data collection

Project Brotherhood will lead the study design, recruitment, focus group facilitation, and dissemination efforts. Quantitative data will be reported descriptively and qualitative data will be analyzed using thematic analysis. For aim one, the quantitative data was collected using a survey developed by the study team that assessed Black mens’ knowledge, attitudes, beliefs, and behaviors related to OPC risk factors and OPC screening. The qualitative data was recorded from focus group sessions run by a trained faciliatator, and assessed participants’ knowledge, attitudes, beliefs, and behaviors related to OPC risk factors and OPC screening. Data were summarized descriptively, and is being used by the advisory panel and research team to refine the communication tool prior to Aim two.

Up to fifty dental students will be recruited to participate in Aim two They will complete a pre-survey and post-survey to assess their comfort levels communicating with Black men about OPCs, and the usefulness of the guide in facilitating those discussions. That data will be used to further develop the tool prior to aim three.

Additionally, dental providers’ perspectives of the tool’s usability and effectiveness in improving their self-efficacy will be assessed. Up to four dental providers treating patients in federally qualified health centers will be recruited to receive training on the use of the tool and document their use of the tool in clinical interactions with Black male patients. Collectively, those providers will recruit up to fifty Black men to participate in the chairside use of the tool and complete a pre-survey and post-survey to asses their increases in knowledge and comfort communicating with dental providers about OPCs. Additionally, the dental providers will be interviewed to provide qualitative feedback of their use of the tool, its feasibility, and usefulness in improving their comfort levels discussing OPCs with Black men.

### Statistical analyses

For aim one, quantitiatve data collected from surveys distributed to Black men to assess knowledge, attitudes, beliefs, and behaviors related to OPC risk factors and screening was analyzed descriptively. Frequency and percentage statistics was tabulated to summarize the data collected. For aim one qualitative data collected from focus groups, thematic analysis was used to identify major themes expressed by Black men regarding their knowledge, attitudes, beliefs, and behaviors related to OPC risk factors and screening. For aim two, pre- and post-surveys will be used to assess dental student’s comfort levels in communicating with Black men about OPCs. Either a paired samples t-test or Wilcoxon singed ranks test will be used to analyze the pre- and post- data, depending on the distribution of the data. For aim three, pre- and post-surveys will be used to asses Black men’s increases in knowledge and comfort communicating with dental providers about OPCs, after interacting with dental providers using the communication tool chairside. Either a paired samples t-test or Wilcoxon singed ranks test will be used to analyze the pre- and post- data, depending on the distribution of the data.

## Discussion

This study aims to develop an OPC communication tool that dental providers can use to effectively communicate OPC risk factors and benefits of prevention to Black men. The long-term goal of this research is for this communication tool to be a modality used to help reduce the long-standing inequities in OPC morbidity and mortaility that Black men in the U.S. experience. There are several strengths in our research approach. Most importantly, we are using a community-based approach to ensure that Black mens’ needs, preferences, and experiences related to oral health communication are used to guide the development of the communication tool. This will help to ensure that the tool is patient-driven rather than provider-driven. While community-based participatory research approach is a major strength of our study, we recognize that the study is being conducted locally among a small sample of Black men residing in Chicago, IL. Therefore, given the diversity of Black men in the U.S., the generalizability of our findings will be limited. However, we view our innovative study as an important first-step from which future research can build upon to test and adapt the OPC communication tool among Black men, dental students, and dental providers in other communities across the U.S. Dissemination of our research findings to public heath, oral health, and medical communities will be critical in ensuring that the tool is validated broadly.

When conducting qualitative research, there is potential of participant response bias and researcher bias. This will be minimized by having researchers use open-ended questions that are engaging and encourage truthful reflection, rather than leading participants to respond in effort to agree with researchers or to align with the study hypothesis. Researchers will also remain neutral in their tone, while refraining from giving their thoughts and opinion about questions being asked. Bias will further be minimized by having researchers complete training and calibration in asking questions to ensure uniformity and maintain rigor. Additionally, data will be analyzed using qualitative methodology aimed to avoid selective interpretation of data among data analysts. Other limitations may occur in study recruitment as for each aim, participants will not be selected randomly so it is possible that those who belong to similar social networks may share similar views. Participants who may be more vocal may also have a deeper interest in the study topic.

## Conclusion

In summary, our research project aims to address the long-standing inequities in OPC outcomes experienced by Black men in the U.S. A community-based participatory research approach will be used to develop a communication tool to enhance dental providers’ communication with Black men related to OPC risk and prevention. The study results will help to inform future research and practice with the ultimate, long-term goal of implementing this tool into clinical practices across the U.S. to improve OPC outcomes in Black men.

## Supporting information

S1 File (PDF)

## References

[1] Weatherspoon DJ, Chattopadhyay A, Boroumand S, Garcia I. Oral cavity and oropharyngeal cancer incidence trends and disparities in the United States: 2000–2010. Cancer Epidemiol. 2015;39(4):497–504. doi: 10.1016/j.canep.2015.04.007 25976107 PMC4532587

[2] National Cancer Institute. Surveillance, Epidemiology, and End Results Program. Cancer Stat Facts: Oral Cavity and Pharynx Cancer. nd. Accessed at https://seer.cancer.gov/statfacts/html/oralcav.html October 14, 2022.

[3] Chiruvella V, Guddati AK. Analysis of Race and Gender Disparities in Mortality Trends from Patients Diagnosed with Nasopharyngeal, Oropharyngeal and Hypopharyngeal Cancer from 2000 to 2017. Int J Gen Med. 2021 Oct 2;14:6315–6323. doi: 10.2147/IJGM.S301837 34629896 PMC8495144

[4] Morse DE, Kerr AR. Disparities in oral and pharyngeal cancer incidence, mortality and survival among black and white Americans. J Am Dent Assoc 2006;137:203–12. doi: 10.14219/jada.archive.2006.0146 16521387 PMC1398075

[5] Tomar SL, Loree M, Logan H. Racial differences in oral and pharyngeal cancer treatment and survival in Florida. Cancer Causes Control. 2004;15: 601–9. doi: 10.1023/B:CACO.0000036166.21056.f9 15280639

[6] Yu AJ, Choi JS, Swanson MS, Kokot NC, Brown TN, Yan G, et al. Association of Race/Ethnicity, Stage, and Survival in Oral Cavity Squamous Cell Carcinoma: A SEER Study. OTO Open. 2019 Dec 10;3(4):2473974X19891126. doi: 10.1177/2473974X19891126 31840132 PMC6904786

[7] Awojobi O, Scott SE, Newton T. Patients’ perceptions of oral cancer screening in dental practice: a cross-sectional study. BMC Oral Health. 2012 Dec 18;12:55. doi: 10.1186/1472-6831-12-55 23249393 PMC3540027

[8] Choi Y, Dodd V, Watson J, Tomar SL, Logan HL, Edwards H. Perspectives of African Americans and dentists concerning dentist-patient communication on oral cancer screening. Patient Educ Couns. 2008 Apr;71(1):41–51. doi: 10.1016/j.pec.2007.11.011 18242933

[9] Howell JL, Shepperd JA, Logan H. Barriers to oral cancer screening: a focus group study of rural Black American adults. Psychooncology. 2013 Jun;22(6):1306–11. doi: 10.1002/pon.3137 22926896 PMC3698600

[10] US Preventive Services Task Force. 2013. Final Recommendation Statement. Oral Cancer: Screening. Accessed at https://www.uspreventiveservicestaskforce.org/uspstf/recommendation/oral-cancer-screening October 14, 2022.

[11] American Academy of Family Physicians. 2022. Clinical Preventive Service Recommendation. Oral Cancer. Accessed at https://www.aafp.org/family-physician/patient-care/clinical-recommendations/all-clinical-recommendations/oral-cancer.html October 14, 2022.

[12] American Cancer Society. 2022. Cancer screening guidelines by age. Accessed at https://www.cancer.org/healthy/find-cancer-early/screening-recommendations-by-age.html#65_or_older October 14, 2022.

[13] Lingen MW, Abt E, Agrawal N, Chaturvedi AK, Cohen E, D’Souza G, et al. Evidence-based clinical practice guideline for the evaluation of potentially malignant disorders in the oral cavity: A report of the American Dental Association. J Am Dent Assoc. 2017 Oct;148(10):712–727.e10. 28958308 10.1016/j.adaj.2017.07.032

[14] Awojobi O, Newton JT, Scott SE. Why don’t dentists talk to patients about oral cancer? Br Dent J. 2015 May 8;218(9):537–41. doi: 10.1038/sj.bdj.2015.343 25952436

[15] LeHew CW, Epstein JB, Kaste LM, Choi YK. Assessing oral cancer early detection: clarifying dentists’ practices. J Public Health Dent. 2010 Spring;70(2):93–100. doi: 10.1111/j.1752-7325.2009.00148.x 19765200

[16] Satcher D. Overlooked and underserved: improving the health of men of color. Am J Public Health. 2003 May;93(5):707–9. doi: 10.2105/ajph.93.5.707 12721126 PMC1447821

[17] Office of Disease Prevention and Health Promotion (ODPHP). nd. Healthy People 2030. Increase the proportion of oral and pharyngeal cancers detected at the earliest state-OH7. Accessed at https://health.gov/healthypeople/objectives-and-data/browse-objectives/oral-conditions/increase-proportion-oral-and-pharyngeal-cancers-detected-earliest-stage-oh-07 September 2, 2022.

[18] Hornik R., Parvanta S., Mello S., Freres D., Kelly B., Schwartz J. S. (2013). Effects of scanning (routine health information exposure) on cancer screening and prevention behaviors in the general population. J Health Commun, 18, 1422–1435. doi: 10.1080/10810730.2013.798381 24083417 PMC4235954

[19] Patrick DL, Lee RS, Nucci M, Grembowski D, Jolles CZ, Milgrom P. Reducing oral health disparities: a focus on social and cultural determinants. BMC Oral Health. 2006 Jun 15;6 Suppl 1(Suppl 1):S4. doi: 10.1186/1472-6831-6-S1-S4 16934121 PMC2147600

[20] Gilbert KL, Ray R, Siddiqi A, Shetty S, Baker EA, Elder K, et al. Visible and invisible trends in Black men’s health: Pitfalls and promises for addressing racial, ethnic, and gender inequities in health. Annu Rev Public Health. 2016;37:295–311. doi: 10.1146/annurev-publhealth-032315-021556 26989830 PMC6531286

[21] Griffith D.M. Centering the margins: Moving equity to the center of men’s health research. American J of Mens Health. 2018;12(5):1317–1327. 10.1177/1557988318773973 PMC6142151 29749300

[22] Smith P. The Oral health status of Black men in the United States: A need for upstream research and multilevel intervention. In: Treadwell H, Evans C, eds. Oral Health in America: Removing the Stain of Disparity. Washington, DC: American Public Health Association; 2019. Chapter 10.

[23] Murray M, Campbell C, Kendall L, Whitt-Glover MC, Watson KS. Perspectives from Project Brotherhood: Facilitating engagement of African American men in research. Prog Community Health Partnersh. 2019;13(5):137–142. doi: 10.1353/cpr.2019.0047 31378744 PMC6954669

[24] Tong A, Sainsbury P, Craig J. Consolidated criteria for reporting qualitative research (COREQ): a 32-item checklist for interviews and focus groups. Int J Qual Health Care. 2007 Dec;19(6):349–57. doi: 10.1093/intqhc/mzm042 17872937

[25] Awojobi O, Newton JT, Scott SE. Pilot study to train dentists to communicate about oral cancer: the impact on dentists’ self-reported behaviour, confidence and beliefs. Br Dent J. 2016 Jan 22;220(2):71–6. doi: 10.1038/sj.bdj.2016.57 26794112

