# Supplementary material for: A community-based participatory protocol to improving communication with Black men about oral and pharyngeal cancers: Research protocol
Source: PLoS One. 2023 Aug 17;18(8):e0288478. doi: 10.1371/journal.pone.0288478 (PMC10434892; doi:10.1371/journal.pone.0288478)
Supplement: S1 File — (PDF) [file pone.0288478.s001.pdf]

APPROVAL

September 1, 2022

Patrick Smith

Dear Dr. Patrick Smith:

On 9/1/2022, the IRB reviewed the following submission:

Type of Review:	Modification / Update
Title:	A Community Engagement Approach to Improving Communication With Black Men About Oral and Pharyngeal Cancers (OPCs)
Investigator:	Patrick Smith
IRB ID:	STUDY2022-0658-MOD001
Funding:	Name: Amer Cancer Soc (IL Div)
Grant Title:	*Illinois Cancer Health Equity Research (I-CHER) Center*
Grant ID:	CHERC-MSI-21-168-01-CHERC-MSI; IP#: 00707325
IND, IDE, or HDE:	None
Documents Reviewed:	None

Please ensure that Dr. Marcus Murray's investigator training is updated before it expires on 10/09/2022. Personnel whose training expires cannot engage in conduct of human subjects' research until their investigator trainings are up to date.

The IRB approved the protocol from 9/1/2022. Before or within 30 business days of study close, whichever is earlier, you are to submit a Continuing Review and required attachments to request continuing approval or closure.

In conducting this protocol, you are required to follow the requirements listed in the Investigator Manual (HRP-103), which can be found by navigating to the IRB Library within the IRB system.

Sincerely,

Office for the Protection of Research Subjects
